# Retraction: Comparing calpain- and caspase-3-mediated degradation patterns in traumatic brain injury by differential proteome analysis

**DOI:** 10.1042/BCJ20050905_RET

**Published:** 2026-05-22

**Authors:** Kevin K.W. Wang, Ming Cheng Liu, Veronica Akle, Wenrong Zheng, Jitendra R. Dave, Frank C. Tortella, Ronald L. Hayes

**Affiliations:** Center for Neuroproteomics and Biomarkers Research, Department of Psychiatry, McKnight Brain Institute, University of Florida, P.O. Box 100256, Gainesville, FL 32610, U.S.A

**Keywords:** calpain, caspase, degradome, high throughput immunoblotting (HTPI), proteomics, traumatic brain injury (TBI)

This article is being retracted from the *Biochemical Journal* at the request of the Editorial Board and its Chair. This action follows the receipt of a notification from a reader, alerting the Editorial Board to multiple concerns surrounding the data presented in the article.

Specifically, this includes the following:

- The Figure 1 western blot also appears as Figure 1D in Bernath et al. 2006 (DOI: 10.1016/j.neurobiolaging.2005.02.013), which has two common authors with this article. The samples are labelled differently – as ‘rat hippocampal lysate’ and ‘rat cortex lystate’ respectively. A corrigendum has been published for Bernath et al. 2006 to disclose the reproduction of the image; however, this article is not cited as part of the corrigendum (DOI: 10.1016/j.neurobiolaging.2025.04.004).
- In Figure 6, bands from lanes 1, 2, 3 and 11 also appear in Figure 7 of Kobeissy et al. 2015 (DOI: 10.1007/s12035-014-8898-z), which has four authors in common with this article. There are contrast differences between the shared images.
- In Figure 7D, the 5^th^ and 6^th^ bands are highly similar after a horizontal flip of the 6^th^ band.

The authors were contacted regarding the concerns. The authors stated that the Figure 1 data was correct, along with the Figure Legend, hence the corrigendum published for Bernath et al. Regarding the similarities with Kobeissy et al. 2015, the authors stated that this was a follow-up experiment and that the data were the same. They acknowledged that the re-use of these images should have been made explicit in Kobeissy et al. The authors dispute the similarity of the western blot bands in Figure 7D; whilst no raw data is available given the age of the article, they maintain that there are pixel-level differences between the bands of the two lanes.

The Editorial Board has assessed all the information presented by the authors and serious concerns remain. The Board therefore stands by the decision to retract; the authors disagree to the retraction.

